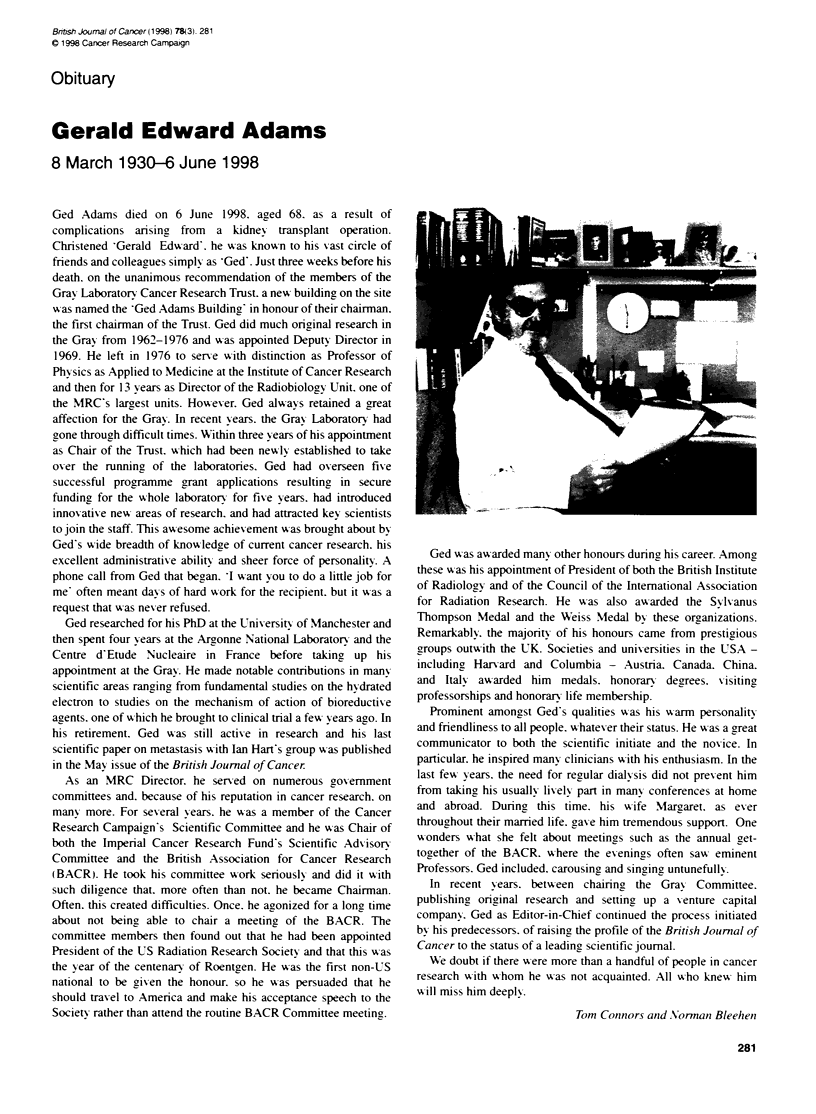# Gerald Edward Adams

**Published:** 1998-08

**Authors:** Tom Connors, Norman Bleehen

## Abstract

**Images:**


British Journal of Cancer (1998) 78(3). 281
© 1998 Cancer Research Campaign

Obituary

### Gerald Edward Adams

### 8 March 1930–6 June 1998

Ged Adams died on 6 June 1998, aged 68, as a result of complications arising from a kidney transplant operation. Christened 'Gerald Edward', he was known to his vast circle of friends and colleagues simply as 'Ged'. Just three weeks before his death, on the unanimous recommendation of the members of the Gray Laboratory Cancer Research Trust, a new building on the site was named the 'Ged Adams Building' in honour of their chairman, the first chairman of the Trust. Ged did much original research in the Gray from 1962–1976 and was appointed Deputy Director in 1969. He left in 1976 to serve with distinction as Professor of Physics as Applied to Medicine at the Institute of Cancer Research and then for 13 years as Director of the Radiobiology Unit, one of the MRC's largest units. However, Ged always retained a great affection for the Gray. In recent years, the Gray Laboratory had gone through difficult times. Within three years of his appointment as Chair of the Trust, which had been newly established to take over the running of the laboratories, Ged had overseen five successful programme grant applications resulting in secure funding for the whole laboratory for five years, had introduced innovative new areas of research, and had attracted key scientists to join the staff. This awesome achievement was brought about by Ged's wide breadth of knowledge of current cancer research, his excellent administrative ability and sheer force of personality. A phone call from Ged that began, 'I want you to do a little job for me' often meant days of hard work for the recipient, but it was a request that was never refused.

Ged researched for his PhD at the University of Manchester and then spent four years at the Argonne National Laboratory and the Centre d'Etude Nucleaire in France before taking up his appointment at the Gray. He made notable contributions in many scientific areas ranging from fundamental studies on the hydrated electron to studies on the mechanism of action of bioreductive agents, one of which he brought to clinical trial a few years ago. In his retirement, Ged was still active in research and his last scientific paper on metastasis with Ian Hart's group was published in the May issue of the *British Journal of Cancer*.

As an MRC Director, he served on numerous government committees and, because of his reputation in cancer research, on many more. For several years, he was a member of the Cancer Research Campaign's Scientific Committee and he was Chair of both the Imperial Cancer Research Fund's Scientific Advisory Committee and the British Association for Cancer Research (BACR). He took his committee work seriously and did it with such diligence that, more often than not, he became Chairman. Often, this created difficulties. Once, he agonized for a long time about not being able to chair a meeting of the BACR. The committee members then found out that he had been appointed President of the US Radiation Research Society and that this was the year of the centenary of Roentgen. He was the first non-US national to be given the honour, so he was persuaded that he should travel to America and make his acceptance speech to the Society rather than attend the routine BACR Committee meeting.

Ged was awarded many other honours during his career. Among these was his appointment of President of both the British Institute of Radiology and of the Council of the International Association for Radiation Research. He was also awarded the Sylvanus Thompson Medal and the Weiss Medal by these organizations. Remarkably, the majority of his honours came from prestigious groups outwith the UK. Societies and universities in the USA – including Harvard and Columbia – Austria, Canada, China, and Italy awarded him medals, honorary degrees, visiting professorships and honorary life membership.

Prominent amongst Ged's qualities was his warm personality and friendliness to all people, whatever their status. He was a great communicator to both the scientific initiate and the novice. In particular, he inspired many clinicians with his enthusiasm. In the last few years, the need for regular dialysis did not prevent him from taking his usually lively part in many conferences at home and abroad. During this time, his wife Margaret, as ever throughout their married life, gave him tremendous support. One wonders what she felt about meetings such as the annual get-together of the BACR, where the evenings often saw eminent Professors, Ged included, carousing and singing untunefully.

In recent years, between chairing the Gray Committee, publishing original research and setting up a venture capital company, Ged as Editor-in-Chief continued the process initiated by his predecessors, of raising the profile of the *British Journal of Cancer* to the status of a leading scientific journal.

We doubt if there were more than a handful of people in cancer research with whom he was not acquainted. All who knew him will miss him deeply.

*Tom Connors and Norman Bleehen*